# Encouraging “Positive Views” of Mental Illness in High Schools: An Evaluation of Bring Change 2 Mind Youth Engagement Clubs

**DOI:** 10.1177/15248399221098349

**Published:** 2022-09-26

**Authors:** Eric H. Fein, Geena Agbangnin, Jovita Murillo-León, Marni Parsons, Rie Sakai-Bismark, Adrienne Martinez, Paola F. Gomez, Bowen Chung, Paul Chung, Rebecca Dudovitz, Moira Inkelas, Sheryl Kataoka

**Affiliations:** 1 Harbor-UCLA Medical Center, Torrance, CA, USA; 2 Bright Star Secondary Charter Academy, Los Angeles, CA, USA; 3 Claremont Graduate University, Claremont, CA, USA; 4 The Lundquist Institute, Torrance, CA, USA; 5 Samuel Merritt University, Oakland, CA, USA; 6 Kaiser Permanente Bernard J. Tyson School of Medicine, Pasadena, CA, USA; 7 University of California, Los Angeles, Los Angeles, CA, USA

**Keywords:** stigma, mental illness, youth engagement, anti-stigma interventions, school health, school mental health services, community-partnered research, adolescent health, Latino youth

## Abstract

“Bring Change 2 Mind” (BC2M) high school clubs may destigmatize mental illness among club members, but clubs’ (1) reach and impact on non-club members at the same school, (2) connection to student help-seeking attitudes, and (3) mechanisms by which they destigmatize mental illness, are unknown. This community-partnered evaluation involved pre/post surveys of predominantly Latino (72%) students at three urban public schools and focus groups and interviews with a sample of club members (n = 26/65, 40%) and all club staff (n = 7, 100%). Multivariate regressions tested relationships between variables. In 84% of the student body responded in the Fall (n = 1,040) and Spring (n = 1,031). Non-club member engagement in BC2M (reach) increased from 25% (Fall) to 44% (Spring) (p < .01). Engagement with BC2M clubs was associated with decreased stigma among members (p < .05) but not non-members (p = .19). Decreased stigma was associated with help-seeking attitudes (p < .01). Possible BC2M mechanisms identified by students and staff include the following: (1) fostering a positive campus climate, (2) normalizing mental health discussions, (3) increasing peer support and help-seeking, and (4) increasing awareness of positive coping behaviors. While BC2M clubs likely reduce stigma for members, effects did not reach non-members, challenging the potential of BC2M clubs as a schoolwide strategy to destigmatize mental health services. Future projects could investigate how to reach non-BC2M members, complement BC2M with other school climate interventions to increase impact, and measure BC2M impact alongside other outcomes relevant to schools, such as academic achievement.

## Introduction

Schools play a key role in providing students with access to mental health services (Ali et al., 2019). Nationally representative reports from the mid-2010s noted that 13.2% of adolescents received mental health services from school annually (Lipari et al., 2013) and 57% of adolescents receiving any mental health services received at least a portion from school (Ali et al., 2019). School staff can identify students dealing with mental health challenges in an accessible, less stigmatizing setting through longitudinal relationships with students (Stephan et al., 2007). Increasing rates of mental illness during the COVID-19 pandemic (Czeisler et al., 2020) place a greater burden on schools to address students’ mental health challenges.

Stigma associated with mental illness is one barrier preventing adolescents from accessing care (Clement et al., 2015). Stigma has been defined as a mark, condition, or status that members of society devalue (Link & Phelan, 2001; Pescosolido & Martin, 2015). Individuals with mental health challenges may avoid seeking care because they fear being stigmatized if others find out they are receiving treatment for mental illness (“treatment stigma”), or may decide not to adhere to therapy due to internalized shame about their condition (“internalized stigma”; Clement et al., 2015; Gulliver et al., 2010).

Effective anti-stigma interventions in youths have typically employed at least one of the following three strategies: (1) promoting mental health *knowledge* (Hadlaczky et al., 2014; Painter et al., 2017), (2) putting individuals in *contact* (through imagined vignettes, videos, or in person) with others who have coped with mental illness successfully (Corrigan et al., 2012), or (3) *engaging* individuals facing their own mental health challenges in programs to help themselves and their peers cope through peer-help skills or activities that affirm those living with mental illness (National Academies of Sciences Engineering and Medicine, 2016; Pescosolido et al., 2020; Sontag-Padilla et al., 2018). These strategies are thought to combat ignorance, increase empathy, and promote positive group identity (Jetten et al., 2018).

### What Is “Bring Change 2 Mind?”

Bring Change 2 Mind (BC2M) high school youth leadership clubs aim to end stigma around mental illness (https://bringchange2mind.org/). BC2M clubs currently exist in hundreds of high schools across the United States. BC2M’s development and current activities are described elsewhere (Ahmad et al., 2020; Goldberg, 2020; Murman et al., 2014; Pescosolido et al., 2020). In brief, the parent not-for-profit organization works with schools to identify student leaders and a school staff member serves as a facilitator. Schools participate *for free*. Once other students volunteer to participate, clubs receive guidance from a national program office and youth advisory board on how to conduct youth-led presentations and school activities to end stigma. Club meetings also provide a safe space to explore mental health topics through peer discussions. Clubs tailor activities as they see fit and require no formal training of leaders. The parent organization’s central office also offers each club a chance to apply for funding, help with organizing activities, and access to a regional youth summit involving teens at participating high schools. In experimental studies, club participants reported reduced stigma (Ahmad et al., 2020; Goldberg, 2020; Murman et al., 2014). (Hereafter, this lessening of stigma toward people with mental illness is described as “positive views” to align with how measures are scored below.) However, significant questions remain about BC2M based on work published thus far.

First, no prior study addresses how BC2M reaches (engages) and affects *non-club* members at participating high schools with a pre/post survey design. Estimating the reach of BC2M is crucial to understanding implementation (Proctor et al., 2011) as a schoolwide strategy to decrease stigma and increase help-seeking among all students. Second, it is unknown if the increase in positive views seen from the BC2M participation translates into improved comfort seeking help for emotional problems. Third, prior peer-reviewed publications do not include interview- or focus group-generated data detailing what takes place in BC2M clubs (Ahmad et al., 2020; Murman et al., 2014). Fourth, the mechanisms by which BC2M increases positive views are unknown (Ahmad et al., 2020; Murman et al., 2014). Testing potential mechanisms could help stigma researchers design useful interventions for high school students and, within the program, strengthen its effectiveness.

Community partnered participatory research (CPPR), a form of community-based participatory research, involves co-planning, consensus building, and power sharing at every step of the research process between community and academic researchers (Chung et al., 2010; Jones et al., 2009). Such partnerships offer numerous benefits, including addressing issues of relevance to under-resourced communities (Thomas et al., 2011); increasing engagement in research (Domecq et al., 2014); and improving translational impact (Khodyakov et al., 2011). Noteworthy examples in mental health services include interventions to address trauma exposure in schools (Ijadi-Maghsoodi et al., 2017; Stein et al., 2002) and depression in adults (Wells et al., 2013).

As part of a nonexperimental, community-partnered program evaluation at three urban public high schools implementing BC2M, with pre- and post-student surveys, we examined BC2M reach and impact for both members and non-members in the following three ways:

1. *Engagement*: Levels of familiarity with and involvement in BC2M activities by BC2M member and non-member students.
2. *Potential effectiveness*: Whether both BC2M and non-BC2M member students would report a greater increase in “positive views” (a decrease in stigma) associated with higher levels of engagement with BC2M activities.
3. Whether positive views would be associated with *help-seeking*.

Through focus groups and interviews with key stakeholders, we also explored student and staff experiences with BC2M to shed light on (1) possible mechanisms and (2) BC2M improvement suggestions.

## Methods

### Approach

This “community-partnered” evaluation involved school staff at three urban public charter high schools; student BC2M leaders at one of the three schools; a research committee that included community and academic research partners affiliated with a public County safety net medical center; and academic partners at a local university. The partnership emerged from a community-academic research engagement conference focused on adolescent emotional well-being (Fein et al., 2021). School staff and partners then decided to study the impact of the new BC2M program they were implementing. Partners met with school staff, student club leaders, and the research committee to design the evaluation. Data were collected by school staff and provided to the research team for the analysis described below. Preliminary results were shared with students and staff in small group classroom meetings at the end of the school year, with reflections incorporated in the discussion. Student leaders at one school and one researcher talked about BC2M and this project on a local radio broadcast (Charles R. Drew University, 2019).

### Setting

In total, participating high schools served about 1,000 students, of whom 84% were Latino, 19% were English language learners, and 92% were eligible for federally subsidized meals. Schools implemented BC2M clubs from August 2018 through June 2019. Counselors at each school served as staff facilitators and solicited student leaders during nonacademic advisory periods early in the 2018 to 2019 school year. Student leaders solicited participation from other students during lunchtime assemblies early in the year. Participation was voluntary. Clubs met at each school approximately one to four times per month throughout year, with students leading meetings. At the end of the year, club participants reported the list of club activities they had completed (Table 1). Individual schools are hereafter referred to as School A, B, and C. An example club discussion topic was “How does your community talk about mental health?” An example club presentation was “Suicide Prevention.” An example schoolwide activity was a “Gratitude Tree” on which students wrote comments of gratitude to other students or staff.

**Table 1:** Bring Change 2 Mind Discussion Topics, Presentations, and School Activities

Club activity	Description	School		
		A	B	C
Club discussions	What is stigma? How is stigma portrayed in current events?		x	x
	Why do people avoid talking about mental illness?		x	x
	Being a friend/family of someone w/mental illness		x	x
	How does your community talk about mental health?	x	x	
	How can you educate your community about mental health?		x	x
	How can you help someone showing signs of mental illness?		x	x
	How does your school handle mental health?		x	
Club presentations	Advocating for change		x	x
	Community mental health			
	Family mental health			x
	Helping friends in need		x	
	Mental health in the media		x	
	Mental illness		x	
	Self-care		x	x
	Stigma		x	x
	Suicide prevention		x	x
	LGBTQ+			
	Beauty standards			
Schoolwide activities	Help students cope with stress/provide self-care Headspace meditation app, students write worries on balloons and pop them, students make stress balls and self-care kits, nap room, self-care flyers, group painting, group board games, silent dance parties, cupcake making	x	x	x
	Encourage students to express themselves about mental health Share stories of students getting through tough times, display art about mental health, school discussion board about mental health topic with sticky notes for student responses, movie night with discussion, students fill happiness jars w/comments about what makes them happy		x	x
	Create a supportive school environment Gratitude tree, supportive letters to open during tough times, recognize students with gives for random acts of kindness, supportive notes to staff from students	x	x	x
	Normalizing sharing about mental illness Mental health walk: Walkers wear colored beads if they know someone dealing with mental illness		x	
	Raise awareness publicly of mental illness stigma Anti-stigma posters at school events		x	x

### Study Design and Participants

Teachers administered anonymous computerized surveys to all students during advisory periods at each school in the Fall (October to December 2018, 2–4 months after club initiation) and Spring (May to June 2019). Students who were absent on the day of administration were not offered another day to complete the survey.

In the Spring of 2019, all BC2M student club members (*n* = 65 total at the three high schools), four staff facilitators (all counselors) at the three schools, and three non-facilitator student wellness counselors were invited to participate in student focus groups (one per school) and staff interviews, respectively, through a scripted, pre-approved, in-person announcement from a research assistant and a scripted, pre-approved email to staff at each high school. Focus groups and interviews took place in the month prior to the last month of the school year. Students and staff were offered monetary gift cards for participating. Students’ parents signed a written informed consent and students signed a written informed assent to participate in focus groups. Staff signed a written informed consent to participate in interviews. Focus groups were conducted at the schools during extracurricular time. Staff interviews were conducted at the school or by phone. A convenience sample of students (School A: *n* = 5/15, B: *n* = 11/25, C: *n* = 10/25) and all student wellness counselors (*n* = 7) at the three schools participated in focus groups and interviews, respectively. Focus groups and interviews followed a semi-structured guide and were recorded and transcribed.

### Quantitative Measures

A full description of measures is included in the Online Supplemental Material. Measures are described briefly here.

#### Measures Collected at Both Fall and Spring

*Measures of positive views* toward people with mental illness included the following: Knowledge about mental illness (e.g., “Talk therapy is a useful way to treat mental illness”), attitudes about mental illness (e.g., “People with mental illness shouldn’t be in regular classes”), and social distance from people with mental illness (e.g., “I would be willing to go on a date with someone with a mental illness”; Ahmad et al., 2020; Murman et al., 2014; Wahl et al., 2011, 2012). To assess BC2M reach, a measure of *how engaged* students were with BC2M was adapted from the following two questions (Sontag-Padilla et al., 2018): “How familiar are you with Bring Change 2 Mind?” and “Compared to other clubs at school, how involved are you with Bring Change 2 Mind?” *Covariates* included self-identified race/ethnicity, school, grade, gender identity, sexual orientation, month of survey administration, and whether the student was a member of BC2M.

#### Measures Collected Only at Spring

Constructs included comfort seeking help from adults at school for emotional problems (“help-seeking”); level of contact with people with mental illness (“contact”), self-reported mental illness (“mental illness”), having met someone with mental illness as part of a BC2M activity, and self-reported knowledge of what mental illness is (“knowledge”).

### Quantitative Data Analysis

Please see the Online Supplemental Material for a full description of analysis procedures. In brief, unadjusted univariate and bivariate analyses of differences between Fall and Spring responses were performed using chi-square tests. To test whether engagement with BC2M was associated with positive views, positive views was regressed on BC2M engagement, time (Fall vs. Spring), and an engagement × time interaction, controlling for BC2M membership, race, gender identity, sexual orientation, grade, and school. Finally, using only data from the Spring, help-seeking was regressed on positive views, controlling for self-reported mental illness, level of contact with people with mental illness, having met someone with mental illness as part of a BC2M activity, and self-reported knowledge of what mental illness is.

### Qualitative Data Analysis

We conducted a cross-sectional, thematic analysis (Braun & Clarke, 2006) guided by the initial steps of Grounded Theory methodology to understand student and staff experiences with BC2M. In the first round of sorting, in vivo quotations were organized by question from the interview guide. These responses were independently coded line-by-line in Microsoft Word. Resulting thematic codes were reviewed by a third member of the study team and discussions were held to reach 100% agreement on final codes and themes. Discussions were held throughout the process and iterative notes were shared through an online document.

## Results

### Participant Demographics

Table 2 illustrates participant demographics at both Fall and Spring. Roughly, the same number of students completed the survey in the Fall (1,040) and Spring (1,031), a response rate of 84% at both times. The sample identified as predominantly Latino (72% in Fall and Spring, *p* = .94), and about one half male (50% in Fall, 51% in Spring, *p* = .78). Roughly, one in 12 students (8% in Fall, 9% in Spring, *p* = .03) identified as lesbian, gay, bisexual, or asexual. Grade level differences between Fall and Spring were not significant (*p* = .44), while school level differences were (*p* < .01).

**Table 2:** Fall and Spring Participant Characteristics

Variable	Fall (*n* = 1,040)		Spring (*n* = 1,031)		*p*
Demographic variables	*n*	%	*n*	%	
Race/Ethnicity					
Latino	744	72	739	72	.94
Non-Latino or multi-racial/ethnic	296	28	292	28	
Gender identity					
Male	523	50	522	51	.78
Female	476	46	471	46	
Non-binary or No response	41	4	38	3	
Sexual orientation					
Heterosexual	852	82	864	84	.03
Lesb./gay/bisex./asex.	84	8	96	9	
Don’t know or no response	104	10	71	7	
Grade					
9	347	33	319	31	.44
10	275	26	262	25	
11	232	22	246	24	
12	186	18	204	20	
School					
A (Total *n* = 496)	328	32	403	39	<.01
B (Total *n* = 290)	256	25	235	23	
C (Total *n* = 496)	456	44	393	38	
Dependent variable	*M* (*SD*)		*M* (*SD*)		
Positive views (1–5)	3.39 (0.5)		3.44 (0.5)		.04
Primary predictor	*n*	%	*n*	%	
BC2M engagement (0–6)					
No engagement (0)	708	69	545	53	<.01
Low engagement (1–3)	248	24	419	41	
High engagement (4–6)	66	6	67	7	
BC2M member	99	10	65	6	<.01
No engagement (0)	11	11	5	8	.11
Low engagement (1–3)	44	45	21	32	
High engagement (4–6)	42	39	43	60	
BC2M non-member	941	90	966	94	<.01
No engagement (0)	697	75	540	56	<.01
Low engagement (1–3)	204	22	398	41	
High engagement (4–6)	24	3	28	3	

*Note.* BC2M = Bring Change 2 Mind.

### Unadjusted Differences in Positive Views and BC2M Engagement and Membership

From Fall to Spring, reported positive views increased significantly (*p* = .04). Overall, engagement in BC2M increased significantly (*p* < .01). For club members only, those reporting high engagement went from 39% in the Fall to 62% in the Spring (*p* = .11). Only 3% of non-club members reported high engagement in BC2M in the Fall and Spring, but the number of non-member students reporting any level of engagement at all jumped from 25% in the Fall to 44% in the Spring (*p* < .01). Keeping engagement as a quantitative variable (and not listed in Table 2), the Pearson correlation coefficient *r* between engagement in BC2M and BC2M membership was .54 (*p* < .01), indicating moderate collinearity.

### Unadjusted Characteristics of Mental Illness, Knowledge, and Contact at Spring Only

Not included in Table 2, in Spring, 9% of students self-reported a mental illness and 29% were unsure. Nearly two-thirds (64%) reported they knew what mental illness is. Only 5% of students were sure they had met someone with mental illness as part of a BC2M club or school activity. On average, students reported a neutral attitude toward comfort seeking help from adults at school for emotional problems (*M* = 3.1, *SD* = 1.1).

### Regression Models of BC2M Engagement as Predictor of Positive Views Over Time

As noted in Table 3, an association between higher engagement with BC2M over time and positive views toward people with mental illness was observed, controlling for BC2M membership, race/ethnicity, gender identity, sexual orientation, grade, and school. The association was significant in the main model with all students (interaction term high engagement × time: β = .21, *p* = .04) and at a greater magnitude when the analysis was restricted to only club members (β = .47, *p* = .04), but not significant when restricting the analysis to only non-club members (β = .18, *p* = .19). In all three models, females reported significantly (*p* < .01) more positive views than males.

**Table 3:** Regressing Positive Views on Interaction With Engagement and Time

Variable	Main model^a^			BC2M members^b^			BC2M non-members^c^		
	β	*SE*	*p*	β	*SE*	*p*	β	*SE*	*p*
Engagement (ref. 0)	–			–			–		
Low engagement	.10	0.04	.00	.03	0.14	.83	.11	0.04	.01
High engagement	.16	0.03	.03	.12	0.17	.48	.05	0.10	.64
Time (dichotomous: Fall = 0, Spring = 1)	.00	0.03	.89	–.22	0.20	.26	.00	0.03	.91
Engagement × time	–			–			–		
Low engagement × time	.00	0.05	.96	.08	0.24	.75	.01	0.05	.84
High engagement × time	.21	0.10	.04	.47	0.22	.04	.18	0.14	.19
BC2M member	–.04	0.04	.37	n/a			n/a		
Latino ethnicity	.07	0.03	.01	.20	0.08	.01	.05	0.03	.06
Gender id. (ref. male)	–			–			–		
Female	.19	0.02	.00	.36	0.08	.00	.17	0.02	.00
Non-binary	.01	0.17	.94	.04	0.52	.94	.01	0.18	.96
Prefer not to say	–.04	0.09	.59	–.15	0.28	.58	.02	0.08	.77
Sexual orientation (ref. heterosexual)	–			–			–		
Lesbian, gay, bisexual, asexual	.20	0.04	.00	.02	0.15	.90	.24	0.04	.00
Other	–.02	0.04	.67	.00	0.12	.99	–.03	0.05	.59
Grade (reference grade 9)	–			–			–		
10	.01	0.03	.77	.21	0.11	.07	–.01	0.03	.80
11	–.03	0.03	.34	.32	0.11	.00	–.06	0.03	.04
12	–.02	0.03	.47	.16	0.17	.37	–.03	0.03	.26
School (reference A)	–			–			–		
B	–.01	0.03	.83	.07	0.10	.47	–.02	0.03	.56
C	–.08	0.02	.00	–.09	0.11	.38	–.08	0.02	.00

*Note.* BC2M = Bring Change 2 Mind.

a Main model: Regressing positive views on engagement, time, engagement × time interaction, BC2M membership, ethnicity, gender, sexual orientation, grade, school. ^b^BC2M members only: Regressing positive views on engagement, time, engagement × time interaction, ethnicity, gender, sexual orientation, grade, school. ^c^BC2M non-members only: Regressing positive views on engagement, time, engagement × time interaction, ethnicity, gender, sexual orientation, grade, school.

### Regression of Positive Views Predicting Help-Seeking From Adults at School

For Table 4, involving data from the Spring, help-seeking attitudes was regressed on positive views, BC2M membership, ethnicity, gender, sexual orientation, grade, school, self-reported mental illness, contact with mental illness, meeting someone with mental illness as part of BC2M, and mental illness knowledge. A significant association was observed between help-seeking and positive views (β = .31, *p* < .01). Other factors significantly associated with help-seeking included engagement; mental illness knowledge; being in the 11th grade; and attending School B. Help-seeking was not associated with BC2M membership, female gender, sexual orientation, or having mental illness when controlling for these other variables.

**Table 4:** Regressing Help-Seeking on Positive Views and Other Spring Covariates

Variable	β	*SE*	*p*
Positive views	.31	0.09	.00
Engagement (ref. 0)	–		
Low engagement	.19	0.08	.02
High engagement	.30	0.19	.11
Contact	–.01	0.01	.54
Met someone with mental illness in BC2M (reference No)	–		
Do not know	.11	0.08	.21
Yes	.13	0.20	.52
Knowledge (reference no)	–		
Do not know	.22	0.14	.11
Yes	.33	0.13	.01
BC2M member	–.09	0.20	.64
Latino ethnicity	.13	0.09	.16
Gender id. (ref. male)	–		
Female	.00	0.07	.98
Non-binary	–.60	0.44	.17
Prefer not to say	–.40	0.26	.22
Sexual orientation (ref. heterosexual)	–		
Lesbian, gay, bisexual, asexual	–.06	0.14	.66
Other	–.09	0.16	.59
Grade (reference grade 9)	–		
10	.16	0.09	.08
11	.30	0.10	.00
12	.01	0.11	.93
School (reference A)	–		
B	.27	0.10	.01
C	–.05	0.08	.54
I have mental illness (ref. No)	–		
Do not know	–.16	0.09	.09
Yes	–.08	0.15	.60

### Possible Mechanisms for BC2M Effectiveness

Through our qualitative analysis, we identified the following four themes reflecting potential mechanisms of BC2M effectiveness: (1) Fostering a positive campus climate, (2) normalizing mental health discussions, (3) increasing peer support and help-seeking, and (4) increasing awareness of positive coping behaviors.

#### Fostering a Positive Campus Climate

All three student focus groups reported that BC2M school activities created positivity on campus. Regarding a particular event consisting of writing encouraging statements across campus with chalk, one member mentioned,

[The event brought] both literally and figuratively . . . brought a lot of color to our campus . . . literally like our blacktop is normally just like dull so in a sense it literally brought like color, but figuratively it just brought like positive vibes.

In a similar program at another school, another member remarked,

I feel like [the encouraging messages] did have a little impact, because . . . it’s like a positive reminder, they could look at it and remind themselves, like, “Oh, I got this” . . . It’s just a nice way of showing that, “hey, people are there to support you.”

Four out of the seven staff reported that club activities created an outlet for positive expression and a place to reduce stress. One counselor talked about a painting night that gave students the opportunity to focus on something other than classes. She reported, “[students’] faces light up because they don’t have that anywhere else on campus where they could just express themselves and be there.” One activity involving a hot cocoa bar for the student body during midterms week was well-received. One counselor mentioned,

[It] definitely add[ed] to school culture positively, because kids feel cared for. They feel pampered, a lot of them, and encouraged. It was a nice surprise to have.

#### Normalizing Mental Health Discussions

Students from two of the three focus groups reported that BC2M created and normalized dialogue about mental health. One club member commented that BC2M “has made it easier to communicate and has lifted a little bit of a filter off of what is this whole mental health thing.” A second student noted,

Because I was part of the club, I was able to tell my friends, and even my family, about what we do, and then what are the common misunderstandings. They were able to learn from it, and they were able to change their habits for good.

A third student spoke about students outside the club being interested in club activities:

[Students] have been thankful for it. Like, “Oh, is this what you guys do? Can you guys tell me more about it?” Then a lot of people just, in general, become more curious about mental health, and then they become more informed about it.

Six of seven staff observed changes in how students within and outside the club spoke about mental health. One counselor spoke about an event held within the club called *Light It Up*: questions were posed to students with the lights out in the room and if that question applied to them, they would turn on the flashlight from their phone. The leader of the club described the environment students reported while participating:

Some of the questions were, “Do you have a family member with a mental illness?” Or, “If you do, do you feel comfortable having the conversation with them?” . . . They were able to get a climate of what everyone else was dealing with, and then kind of have a conversation about it afterwards. I think that was the most impactful because they were able to see that they were not alone in their struggles.

#### Increasing Peer Support and Seeking Help

All three student focus groups noted that BC2M led to greater peer support on campus and encouraged seeking help. One member reflected,

I have a friend who is never really the one to speak out about stuff that is going on . . . I was like telling what we were doing during clubs and what she can do to help herself, or like what I can do to help her personally. And then when I would text her, like, “Oh, are you all right? Do you need help with anything? I’m here.” Before she would just be like, “No, I’m okay, and I got this. Nothing is wrong with me.” But now that I told her everything about the club, what she can do, she’s more open about talking about it. In that way, she’s gotten more open. She’s told me that me helping her through everything has actually helped her going through what she’s going through.

Six of the seven staff spoke about club members checking up on other classmates and students seeking help from counselors because of their involvement. One mentioned,

[The club members] notice certain behaviors in kids and they’ll text me. They’ll be like, “Miss, so-and-so is not having—like they’re not okay right now. Can you go check in on them?” So, they watch out for other people.

#### Increasing Awareness of Positive Coping Behaviors

Two of the three student focus groups conveyed that BC2M clubs promoted mental health awareness in the form of self-care and resources. One club member spoke about an activity making stress balls, stating,

Stress balls are supposed to be something you use . . . (to) cope with whatever it is that you are stressing with . . . in a way it allowed for those who maybe didn’t know what a stress ball was to now have a resource for them.

A club member from another school spoke specifically about the presentations during an advisory period saying, “there were also helpful links that if you were going through this, you can go to one of these sites.” Another member discussed that the club gives students knowledge they may not receive elsewhere about mental health, stating, “We’re helping them by educating them, and that’s always good because we’re not exposed to . . . certain things.”

Five of the seven staff perceived that club members were more aware of their own mental health and promoted self-care within club events. One staff member noticed a shift in the students during counseling sessions, saying,

They’re already coming to me with strategies that they’ve heard and they know as opposed to just coming to me. I think they’re just a little bit more informed.

Table 5 summarizes improvement suggestions for BC2M clubs. The following four improvement goals were identified: Engaging more members of the school community; having more time and more guidance to plan club activities; providing members of the school community with more knowledge, skills, and awareness of resources; and determining how BC2M activities affected students. Example strategies to achieve each goal, respectively, included the following: Engaging parents with a parent mental health night; giving students more time after school to conduct club activities; giving staff more tools to deal with students with mental health challenges; and end-of-year student reflections on club activities.

**Table 5:** Improvement Goals and Strategies Identified by Students and Staff

Goals	Strategies to achieve goals
1. Engage more members of school community -Boys -Different ethnic backgrounds -Different grade levels -Multiple staff as club facilitators -Administrators -Parents	-Parent liaison from the club -Mental health awareness training -Parents mental health night -Advertise who is in the club and what it does, nonacademic nature -Enlist buy-in from administrators and teachers -Humanize administrators and teachers by letting them share personal stories about mental health challenges, showing up at BC2M events
2. More time and more guidance to plan BC2M club activities	-Give students more time, after school -Set goal of one schoolwide activity per month -Help students get organized
3. Provide members of school community more knowledge, skills, and awareness of local mental health resources	-Mental health awareness training for students -Employ club members as peer ambassadors to share resources -Give staff tools to deal with students with mental health challenges -Pair fun club activities with youth-led discussions -Discuss benefits of counseling with parents -Discuss benefits of self-care with students
4. Determine how club activities affected students	-End of year student reflection -Ask students what info they want and match club activities to this

Regarding engaging (reaching) more members of the school community, students, and staff mentioned: specific outreach to boys and students with different ethnicities, involving multiple staff to remove the burden from any one staff member, and identifying a parent liaison between the club and other parents to help conduct a parent mental health night. They felt that allowing staff to share with students about mental health challenges they had overcome would humanize them and further destigmatize mental health. Students also advocated for increased advertising of the club as a nonacademic group allowing students to socialize.

## Discussion

This is the first peer-reviewed evaluation of BC2M to (1) examine the reach and impact of BC2M on *both* BC2M members *and* non-members in a pre/post survey design, (2) examine the potential effect of BC2M participation on help-seeking behavior, and (3) explore possible mechanisms by which BC2M increases positive views toward people with mental illness. Our non-experimental evaluation—involving three high schools newly implementing the club with predominantly under-resourced, Latino students—found that while club members reported greater positive views, the clubs did not significantly affect positive views of other students at the school.

For BC2M members, given the flexibility schools have in implementing BC2M, repeated positive results are noteworthy and increase confidence in the validity and generalizability of prior experimental results. Student and staff focus groups and interviews coalesced around four ideas about how BC2M may increase positive views, by (1) fostering a positive campus climate, (2) normalizing mental health discussions, (3) increasing peer support and help-seeking, and (4) increasing awareness of positive coping behaviors. While other anti-stigma interventions are based on contact with individuals dealing with mental illness to increase empathy (Chen et al., 2016; Corrigan et al., 2012), the mention of students feeling supported within their school does not necessarily depend on contact with people with mental illness. Perhaps, if students perceive that those around them will support them in general, they will feel more comfortable discussing their problems and asking for help (Townsend et al., 2017).

For non-club members, non-significant findings likely relate to the fact that only 3% of non-member students reported high engagement in both Fall and Spring, suggesting poor reach. Findings thus challenge the use of BC2M as an effective *schoolwide* strategy to reduce stigma, but improvement suggestions noted in Table 5 offer testable engagement strategies in the future. Also of note, in Spring 2019, students and staff were not aware of BC2M material with culturally specific and relevant BC2M material for racial or ethnic minority students, which they identified as a major barrier to engagement. (At the time of this writing, BC2M does offer material on racial trauma, Native Americans, allyship, Hispanic and Latino populations, and men’s health (Bring Change 2 Mind, n.d.).)

Concerning the participatory approach, staff attribute the high survey response rate to recruiting efforts around a survey over which they perceived ownership and carved out time from the day for students to respond. Students and staff appreciated the opportunity to speak their minds. Researchers appreciated the opportunity to study a topic relevant to under-resourced schoolchildren, and within the evaluation, contextual information about BC2M implementation otherwise not available to them. Staff, at times, felt the project competed with other demands on their time.

### Limitations

Observational design, lack of a control group, and the use of anonymous surveys that were not linked at the individual level over time (as requested by our school partners) limit causal inference. Clubs at each school did not provide fidelity checklists detailing components of activities performed. We did not measure potential confounders, including stigma expressed by peers, parents, or staff. Moreover, our Fall survey was completed 2 to 4 months after BC2M implementation had started, possibly blunting changes that we could measure over time. Social desirability bias (not measured) may limit the accuracy of student report. We also did not recruit students who were *not* members to focus groups to discuss reach.

## Implications for School Mental Health Research and Practice

Based on this evaluation and the previous two BC2M studies (Ahmad et al., 2020; Murman et al., 2014) and a published, non-peer reviewed report (Goldberg, 2020), BC2M remains a *promising*, *inexpensive* strategy for high schools to engage student members in reducing stigma and seek out evidence-based mental health services.

Future research could address how to engage more individuals in club activities, with strategies for specific groups. For instance, to engage students with diverse ethnicities, BC2M leaders could host presentations with members of specific ethnicities to align with holidays that are celebrated in those communities. Parents could be engaged as liaisons between the club and other parents and host activities at parent engagement events. Youth advisory boards, parents, and staff could partner with investigators to study which activities engage the most students and are most impactful, and what factors are most important to measure (e.g., stigma, mental health awareness, access to care, symptomatology, school climate, academic indicators, and differences in findings by demographic groups). BC2M will likely have a greater impact if implemented as part of a broader, evidence-based curriculum such as Mental Health First Aid (Hadlaczky et al., 2014). To help school districts practically assess reach and impact of BC2M and other campus initiatives with high response rates, districts could add a short questionnaire to beginning and year-end mandatory student surveys.

In conclusion, given the prevalence of mental health challenges and the key role that schools occupy in adolescent students’ lives, schools can provide students with a pivotal point of access to mental health services. Decreasing the stigma of mental illness in schools thus could help students realize this access, leading to broad public health impact. Bring Change 2 Mind secondary school youth leadership clubs offer North American schools an inexpensive, promising, and easily available option to reduce stigma among club members. Unfortunately, in our study, involving three high schools with predominantly under-resourced Latino students, the impact on stigma among students outside the clubs was not significant, even though nearly half the student body at the three schools reported some involvement in club activities by the end of the school year. Given the positive findings and benefits of BC2M that student members voiced in this and other BC2M studies, further research into how to improve the reach and effectiveness of BC2M is warranted. Investigators could study how to augment BC2M effectiveness by pairing it with other evidence-based approaches; how to engage more students, staff, and parents in BC2M activities; and how BC2M affects other relevant outcomes, like academic achievement, mental illness symptoms, and school climate.

## Supplemental Material

sj-do-1-hpp-10.1177_15248399221098349 – Supplemental material for Encouraging “Positive Views” of Mental Illness in High Schools: An Evaluation of Bring Change 2 Mind Youth Engagement Clubs Supplemental material, sj-do-1-hpp-10.1177_15248399221098349 for Encouraging “Positive Views” of Mental Illness in High Schools: An Evaluation of Bring Change 2 Mind Youth Engagement Clubs by Eric H. Fein, Geena Agbangnin, Jovita Murillo-León, Marni Parsons, Rie Sakai-Bismark, Adrienne Martinez, Paola F. Gomez, Bowen Chung, Paul Chung, Rebecca Dudovitz, Moira Inkelas and Sheryl Kataoka in Health Promotion Practice

sj-docx-2-hpp-10.1177_15248399221098349 – Supplemental material for Encouraging “Positive Views” of Mental Illness in High Schools: An Evaluation of Bring Change 2 Mind Youth Engagement Clubs Supplemental material, sj-docx-2-hpp-10.1177_15248399221098349 for Encouraging “Positive Views” of Mental Illness in High Schools: An Evaluation of Bring Change 2 Mind Youth Engagement Clubs by Eric H. Fein, Geena Agbangnin, Jovita Murillo-León, Marni Parsons, Rie Sakai-Bismark, Adrienne Martinez, Paola F. Gomez, Bowen Chung, Paul Chung, Rebecca Dudovitz, Moira Inkelas and Sheryl Kataoka in Health Promotion Practice

sj-dta-3-hpp-10.1177_15248399221098349 – Supplemental material for Encouraging “Positive Views” of Mental Illness in High Schools: An Evaluation of Bring Change 2 Mind Youth Engagement Clubs Supplemental material, sj-dta-3-hpp-10.1177_15248399221098349 for Encouraging “Positive Views” of Mental Illness in High Schools: An Evaluation of Bring Change 2 Mind Youth Engagement Clubs by Eric H. Fein, Geena Agbangnin, Jovita Murillo-León, Marni Parsons, Rie Sakai-Bismark, Adrienne Martinez, Paola F. Gomez, Bowen Chung, Paul Chung, Rebecca Dudovitz, Moira Inkelas and Sheryl Kataoka in Health Promotion Practice
