# Supplementary material for: Encouraging “Positive Views” of Mental Illness in High Schools: An Evaluation of Bring Change 2 Mind Youth Engagement Clubs
Source: Health Promot Pract. 2022 Sep 26;24(5):873–85. doi: 10.1177/15248399221098349 (PMC10481624; doi:10.1177/15248399221098349)
Supplement: sj-docx-2-hpp-10.1177_15248399221098349 – Supplemental material for Encouraging “Positive Views” of Mental Illness in High Schools: An Evaluation of Bring Change 2 Mind Youth Engagement Clubs [file sj-docx-2-hpp-10.1177_15248399221098349.docx]

**Quantitative Measures**

***Measures Collected at Both Fall and Spring***

**Measures of Positive Views: Knowledge; Attitudes; and Social Distancing.** Measures used were chosen to match measures used in previous studies of BC2M: Knowledge about mental illness (e.g., “Talk therapy is a useful way to treat mental illness”); attitudes about mental illness (e.g.: “People with mental illness shouldn’t be in regular classes”); and social distance from people with mental illness (e.g.: “I would be willing to go on a date with someone with a mental illness”) (Ahmad et al., 2020; Murman et al., 2014; Wahl et al., 2011, 2012). Each of these scales was shortened to four questions given concerns by school staff of burden to students. The items selected were those with the largest change from Fall- to Spring- in prior studies using the full scales. Our primary outcome, “positive views,” was created by averaging the results of 4 knowledge, 4 attitudes, and 4 social distancing questions together. Response options ranged on a 5-point Likert scale from strongly disagree to strongly agree, scored such that 1 corresponded with the most negative views and 5 corresponded with the most positive views toward people with mental illness. Taken together, the internal consistency Cronbach alpha of the 12-item “positive views” scale was 0.73 in the Fall and 0.77 at Spring.

**Engagement: Familiarity and Involvement with BC2M.** Engagement in BC2M was scored as a sum of two questions adapted from an evaluation of a university-based anti-stigma youth engagement group (Sontag-Padilla et al., 2018): “How familiar are you with Bring Change 2 Mind?” and “Compared to other clubs at school, how involved are you with Bring Change 2 Mind?” Each was scored 0-3 based on a Likert scale with higher scores corresponding to more familiarity or involvement with BC2M. The two questions were summed and three categories of engagement were created: no engagement (score=0); low engagement (score=1-3); and high engagement (score=4-6). Internal consistency Cronbach alpha of the two items together was 0.77 in Fall and 0.73 in Spring. As a sensitivity analysis, all analyses performed were repeated using only BC2M familiarity and only BC2M involvement: Changes in statistical significance and effect sizes were not observed. Covariates included self-identified race/ethnicity, school, grade, gender identity, sexual orientation, month of survey administration, and whether the student was a member of BC2M.

***Measures Collected Only at Spring***

Constructs included: comfort seeking help from adults at school for emotional problems (“help-seeking”); level of contact with people with mental illness (“contact”), self-reported mental illness (“mental illness”), having met someone with mental illness as part of a BC2M activity, and self-reported knowledge of what mental illness is (“knowledge”).

*Help-Seeking* was rated from 1-5 on a Likert scale, indicating strong disagreement (1) to strong agreement (5) with a statement generated by school staff: “I am comfortable seeking help from an adult at school for an emotional problem”.

*Contact* was a summed scale of 7 items validated in adolescents to assess the level of social contact a person has with people with mental illness (Corrigan et al., 2005). Sample items include: “I have watched a show that included a person with mental illness”, “I have been in a class with a person with mental illness”, and “I live with a person with mental illness”. Possible responses were scored as 0 (False), 1 (Don’t know), or 2 (True) and added together, such that higher scores correspond to higher levels of contact with a person with mental illness. Self-reported mental illness (“mental illness”) was assessed as an optional item, to facilitate students’ sense of psychological safety, using the last (eighth) item from the level of contact scale:^18^ “I have a mental illness” with the same response options. Response rate for this item was 959/1,031, or 93%. the Cronbach alpha for the level of social contact scale was 0.71. Students were also asked if they met someone with mental illness as part of a BC2M activity, with response options “no” (0), “do not know” (1), “yes” (2).

*Knowledge* was measured by level of agreement with the student-generated statement “I know what mental illness is,” with three possible responses (0=False, 1=Don’t know, 2= True).

**Quantitative data analysis**

Statistical analysis was performed using Stata version 15 (StataCorp LP, College Station, Texas). Unadjusted univariate and bivariate analyses were performed using chi-squared tests to examine differences in all variables in the Fall vs Spring, including changes in engagement for different groups of students between the Fall and Spring. Individual differences from Fall to Spring were not conducted since schools were not able to link data at the individual level. Statistical significance was defined as p < .05.

To test whether engagement with BC2M was associated with positive views, a robust OLS regression using heteroskedastic error terms to account for clustering was performed that regressed positive views towards on BC2M engagement and time, plus an interaction term between BC2M engagement and time (Fall vs. Spring), controlling for BC2M membership, race, gender identity, sexual orientation, grade, and school. A second regression analysis was conducted stratifying by BC2M membership. Since cross-sectional, and not longitudinal, data were collected, stratified regressions were conducted by regressing positive views on time and other covariates, but restricting each regression to students reporting no engagement, low engagement, and high engagement separately.

Only data from the follow up survey administered in the Spring were used to test whether higher positive views would be associated with help-seeking. Robust OLS regression of positive views on engagement, BC2M Membership, race, gender, sexual orientation, grade, school, and self-reported mental illness was performed. Level of contact with people with mental illness, having met someone with mental illness as part of a BC2M activity, and self-reported knowledge of what mental illness is were then added into a follow-up regression as covariates. Finally, help-seeking was regressed on positive views and these other variables.
